# Production kinetics of polyhydroxyalkanoates by using *Pseudomonas aeruginosa* gamma ray mutant strain EBN-8 cultured on soybean oil

**DOI:** 10.1007/s13205-016-0452-4

**Published:** 2016-06-23

**Authors:** Sharjeel Abid, Zulfiqar Ali Raza, Tanveer Hussain

**Affiliations:** Chemistry Research Laboratory, National Textile University, Faisalabad, 37610 Pakistan

**Keywords:** PHAs, Polyhydroxyalkanoates, *Pseudomonas aeruginosa*, Shake flask, Soybean oil

## Abstract

The purpose of present study was to optimize polyhydroxyalkanotes (PHAs) production in a gamma ray mutant strain of *Pseudomonas aeruginosa* grown on soybean oil in minimal salts media under shake flask conditions. The production kinetics was studied by sampling on daily basis for 6 days to investigate the best conditions for PHAs production like biomass estimation, carbon source utilization and PHAs yield. The PHA accumulation was observed up to 50.27 % (w/w) of cell dry mass. The *Pseudomonas* species synthesized medium chain length PHA copolyester as per identified by LCMS and confirmed by FTIR spectroscopy. The ESI–MS analysis exhibited the major polyhydroxybutyrate with a molecular mass of *m/z* 448.5.

## Introduction

Polyhydroxyalkanoates (PHAs) are one of the major candidates to replace petroleum derived polyesters due to their extraordinary properties and they have one of the widest range of molecular masses from 1 × 10^4^ to 1 × 10^6^ amu (Chen 2010). These are high molecular mass biological polyesters synthesized in many living organisms. The properties of PHAs cover wide range from materials like poly (propylene) to elastomeric materials. Their diverse properties are either due to the distant variation between ester linkages or the length of pendant groups extending from back bone (Williams et al. 1999). Properties of conventional plastics as well as PHAs can be tailored as desired from packing materials to highly elastic ones like coatings but the PHAs have the edge of being bio-degradable as well as they are produced from the sources which are renewable (Loo and Sudesh 2007).

Plants and different types of microorganisms can produce PHAs but cell dry mass (CDM) of less than 10 % (w/w) is observed in plants, however bacteria can accumulate as high as 90 % (w/w). This low accumulation in plants is due to the fact that greater than 10 % (w/w) accumulation generally has a negative impact on plant growth (Verlinden et al. 2007; Tan et al. 2014). On the other hand, more than 250 different bacterial strains can produce PHAs under various conditions and they generate PHAs with different levels of quality, yield and efficiency (Pollet and Averous 2011).

Conventional plastics produced from petrochemical sources are associated with the main issues of disposal and production from limited resources of petrochemicals resulting in the accumulation of large waste in the environment (landfill and sea water). Occurrence of PHAs was reported by Lemoigne in 1920s but the main reason of preferring conventional petrochemical based plastics over bioplastics was the low cost of formers and high cost of latters (Verlinden et al. 2007). One of the major factors for high cost of PHAs is the high cost of carbon source the researchers, therefore, are trying to explore some inexpensive sources like waste frying oils to reduce the cost associated with PHAs production (Lee et al. 2008). *Pseudomonas* sp. has been reported to produce 37.34 and 23.52 % (w/w) PHAs from corn oil and waste frying oils, respectively (Song et al. 2008).

Agriculture and food industries dispose off a large amount of carbon and other nutrients rich waste which can be used for the economical production of PHAs as this waste can be utilized as renewable source by many microorganisms hence giving more ecological and attractive alternative usage than disposal of this waste into the environment (Song et al. 2008; Chee et al. 2010; Prasad and Sethi 2013). Plant oils (like palm oil, soybean oil, sunflower oil etc.) are preferred to sugars as sole carbon source for PHAs production as they are cheaper and produce more quantity of PHAs per gram of carbon source, for instance, 0.3–0.4 g of PHAs have been reported by 1 g of glucose; on the other hand, plant oils yield 0.6–0.8 g of PHAs per gram of oil. This higher production could be attributed to the more carbon content per unit mass of plant oils as compared to sugars (Chee et al. 2010). The higher catabolism rate of lipid substrates (beta-oxidation at lipids) than other substrates (Muhr et al. 2013a, b) and the fact that these saturated and unsaturated fats can easily be metabolized by microorganisms to synthesize PHAs (Koller et al. 2012).

For the batch production of PHAs in bacterial cells, two modes are normally used. In the first, accumulation of PHAs starts at the exponential phase and continues until the late stationary phase and is known as one stage batch cultivation. In the second, cultivation of bacteria is done in a separate growth favoring environment and when the cells are enriched, they are transferred to the PHAs accumulation phase where nutrient depletion is used for this purpose (Kumar and Abe 2010). For the survival of cells when bacteria face starvation, fluctuations in environmental conditions, giving response to sporulation or encystment, bacteria store important nutrients where PHAs are one of the major such storage compounds (Haba et al. 2007).

For the production of PHAs within bacterial cells, the carbon sources are comprehended and then transmuted into hydroxyalkanoates followed by polymerization into PHAs and stored in cell cytoplasm as water insoluble granules. These granules appear as spherical particles with clear boundaries and are electron transparent. These PHAs in granules form are kept in amorphous state in vivo otherwise if crystallized these granules cannot serve as storage compound for the host bacterial cell producing it (Loo and Sudesh 2007). *Pseudomonas* sp. is one of the most studied microorganism for PHAs accumulation (Jiang et al. 2008). *P. aeruginosa* generally produce medium chain length (mcl) PHAs (Soberon-Chavez et al. 2005a). Various studies on *Pseudomonas* species showed that different carbon sources like alkanes, alkene, carboxylic acids, alcohols, carbohydrates and others could be used for mcl PHAs production (Silva-Queiroz et al. 2009). *P. aeruginosa* could produce PHAs and rhamnolipids simultaneously when subjected to nitrogen limited conditions (Marsudi et al. 2008).

The EBN-8 gamma ray mutant of *P. aeruginosa* has previously been studied for enhanced production of biosurfactant (Raza et al. 2014) but has not been studied for the production of PHAs when grown on soybean oil. The aim of present study is to investigate production kinetics of PHAs by using a *Pseudomonas aeruginosa* mutant strain grown on soybean oil as carbon source under shake flask conditions. The synthesized polymer was isolated, purified and chemically analyzed for identification.

## Materials and methods

### Materials

Soybean cooking oil was purchased from Hamza vegetable oil refinery and ghee mills (Pvt.) Ltd, Lahore-Pakistan. All the chemicals used in this study were of analytical grade. KH_2_PO_4_, K_2_HPO_4_, FeSO_4·_7H_2_O, chloroform, methanol and acetone were purchased from Sigma-Aldrich^®^. CaCl_2·_2H_2_O and MgSO_4·_7H_2_O were purchased from Riedel-de Haen^®^, and NaNO_3_ from Merck^®^. Petroleum ether was supplied by Fisher^®^ chemicals.

### Microorganism

The gamma ray induced mutant strain of *Pseudomonas aeruginosa* designated as EBN-8 (Iqbal et al. 1995) used in this study was kindly donated by National Institute for Biotechnology and Genetic Engineering (NIBGE), Faisalabad-Pakistan. The inoculum was prepared by suspending the cells of bacteria in normal saline (0.89 %, w/v, NaCl) and set an optical activity of 0.7 at 660 nm. This suspension was used as inoculum during the present study.

### Shake flask experiment

For the shake flask experiments, following recipe (g/l) was used for minimal salts media prepared in distilled water: KH_2_PO_4_ (0.07), K_2_HPO_4_ (0.13), NaNO_3_ (0.2), MgSO_4·_7H_2_O (0.03), CaCl_2·_2H_2_O (0.01) and FeSO_4·_7H_2_O (0.0001). The pH-value of the solution was adjusted to 7 when required and 100 ml of solution were shifted in separate 250 ml Erlenmeyer flasks and sterilized at 121 °C for 15 min in an autoclave (WiseClave^®^; WAC-60, Korea). Three concentrations of soybean oil i.e., 1, 2 and 3 % (v/v) were used. The inoculum (1 %, v/v) was added and the flasks were placed on an orbital shaker (WiseShake^®^; SHO-2D, Daihan, Korea) at 150 rpm and 37 ± 1 °C.

Sampling was carried out up to 6th day of incubation and on each day different process attributes were monitored to optimize the production conditions of PHAs including: carbon source utilization (w/w %), biomass yield (g/l) and PHA yield (g/l). The experiments were conducted in triplicate and the results reported are the average of three concordant readings.

### Carbon source utilization

For the optimization of PHAs production, carbon source consumption is one of the important parameters to study as it is one of the cost determining factors. For carbon source utilization calculations, cell free culture broth (CFCB) was used. An aliquot of 50 ml of CFCB was washed with 1:2 (v/v) petroleum ether and the organic layer was separated by using a separating funnel. The organic solvent layer containing the soybean oil was concentrated on a rotary evaporator machine (Strike^®^ 202, STEROGLASS, Italy) at 60 °C to a constant mass. The carbon source utilization was calculated by the Eq. 1:

1 $${\text{Carbon source utilization (w/w}}\,\%) = \left( {\frac{{{\text{Initial oil} - \text{Residual oil}}}}{\text{Initial oil}}} \right) \times 100.$$

### Biomass estimation

An aliquot (20 ml) from the sample flask was centrifuged at 10,000 rpm and 4 °C by using a refrigerated centrifuge machine (MSE, Harrier 18/80R, England) to collect the cell pellet. The pallet was washed with *n*-hexane to remove any oil traces adhered to the cells and re-suspended using a vortex shaker (WiseMix^®^; VM-10 by Daihan, Korea) and filtered through Whatman^®^ Sigma-Aldrich filter paper. The filter paper was pre-weighed before filtration and after filtration to a constant dry mass using moisture analyzer (UniBloc moisture analyzer, model MOC63u by SHIMADZU, Japan). The cell dry mass (CDM) was calculated by using Eq. 2:

2 $${\text{CDM}} = W_{2} {-}W_{1} ,$$

where, *W*_2_ is final dry mass of filter paper including cell pallet and *W*_1_ is initial dry mass of filter paper without pallet.

### Extraction of PHA from cells

Solvent extraction method was used for the extraction of PHAs from the cells of *P. aeruginosa* as this method does not degrade the polymer (Jacquel et al. 2008). The cells were collected by centrifugation at 10,000 rpm and 4 °C for 10 min. Acetone washing was used to remove any organics from the cell surface (Brown 1996). The acetone washed cells were subjected to re-washing with an anionic surfactant Triton X-100 to make cells permeable and extract the organelles (Koley and Bard 2010). Then, the cells were freeze dried for overnight using Heto Power Dry LL1500 freeze dryer (Thermo Electron Corporation, USA). These cells were then re-suspended to 50 ml chloroform, shifted in a 250 ml flask and placed on the orbital shaker at 180 rpm at 30 °C for 24 h for the extraction of bio-polymer. Then, the cell debris was removed through Whatman^®^ filter paper and the filtrate was concentrated using the rotary evaporator. Then, chilled methanol was added (1:3) drop wise to precipitate PHA polymer (Tan et al. 2014). A milky precipitate containing solution (chloroform and methanol) was rotary evaporated and the concentrate containing the PHA polymers was weighed to a constant mass at 60 °C (Jiang et al. 2008).

### Production kinetics

Production kinetics of fermentation was studied by calculating the product yield with respect to substrate consumption *Y*_P/S_ (g/g), product yield with respect to biomass *Y*_P/X_ (g/g), biomass yield related to substrate consumption *Y*_X/S_ (g/g) and volumetric productivity *P*_V_ (g/l/h) of the culture media (Aiba et al. 1973).

### Fourier transform infrared spectroscopy (FTIR)

FTIR (Bruker, Tensor 27) was used to analyze the produced PHA polymer as it is one of the most used characterization technique for identification of polymers (Chalmers et al. 1996; Koller and Rodríguez-Contreras 2015).

## Liquid chromatography–Mass spectroscopy (LC–MS)

The PHB (or PHA) matrix was prepared in chloroform for its ESI chemical analysis on a double focusing mass spectrometer (LTQ XL, Thermo electron Corporation USA) both in positive and negative ion modes equipped with a liquid chromatograph (Finnigan Surveyor, Thermo electron Corporation, USA) at the running conditions of: capillary temperature 280 °C, sheath gas flow rate 25, auxiliary gas flow rate 5, tube lenz 70 V, source voltage 3.5 kV and flow rate 10 μl/min.

## Results and discussion

Extensive research is being performed on PHAs due to many advantageous properties of the bio-polymer over conventional petrochemical based plastics. *Pseudomonas* species which belong to the Gram-negative bacteria are well known to accumulate PHAs when grown on different substrates under favorable growth conditions. One of the major cost factors in bacterial fermentation is carbon source (Sudesh 2012). For the optimization of present study carbon source utilization, biomass formation and PHAs production has been calculated.

### Carbon source utilization

**Fig. 1:**
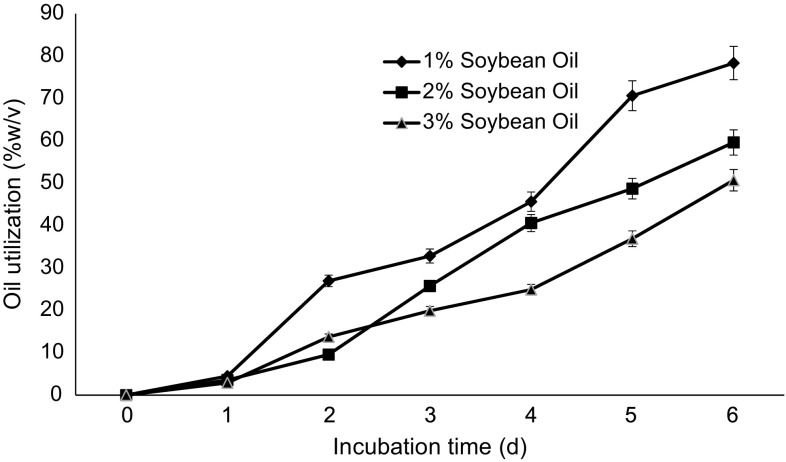
Carbon source utilization during incubation under shake flask conditions

The amount of carbon source utilized was checked by measuring the residual oil contents in the CFCB. The carbon source utilization results are shown in Fig. 1. *P. aeruginosa* has been reported for the production of biosurfactant (Haba et al. 2000; Hori et al. 2002; Marsudi et al. 2008; Soberon-Chavez et al. 2005b). The EBN-8 gamma ray mutant used in this study had also been reported to produce of rhamnolipids and with each passing day of incubation, an increased production of rhamnolipids was reported which resulted in a decrease of surface tension of the culture broth (Raza et al. 2006). The reduced surface tension attributed more access of bacteria to the carbon source which was not easily accessible on first day of incubation when no rhamnolipids were produced. Thus, the carbon source utilization increased from 1st day of incubation to 6th day of incubation due to more rhamnolipids production as well as increased concentration of bacteria day by day on all three concentrations but maximum utilization of soybean oil was 78.35 % (w/w) at 6th day of incubation when 1 % (v/v) soybean oil was used as sole carbon source. When 2 and 3 % (v/v) of soybean oil was used, maximum utilizations were 59.61 and 50.7 % (w/w), respectively. This could be attributed to the fact that initial concentration of the soybean oil varied hence the utilization of soybean oil too. The concentration of soybean oil available to the 2 and 3 % (v/v) cultures were high resulting in less oil utilization overall than 1 % (v/v) cultures. Irrespective of the concentration of the soybean oil used for growth of bacteria, the trend of increased utilization was observed which is shown in Fig. 1; as the number of bacterial cells increased, the requirement of carbon source for growth increased and production of rhamnolipids increased access of bacteria to hydrophobic carbon source.

### Biomass formation

**Fig. 2:**
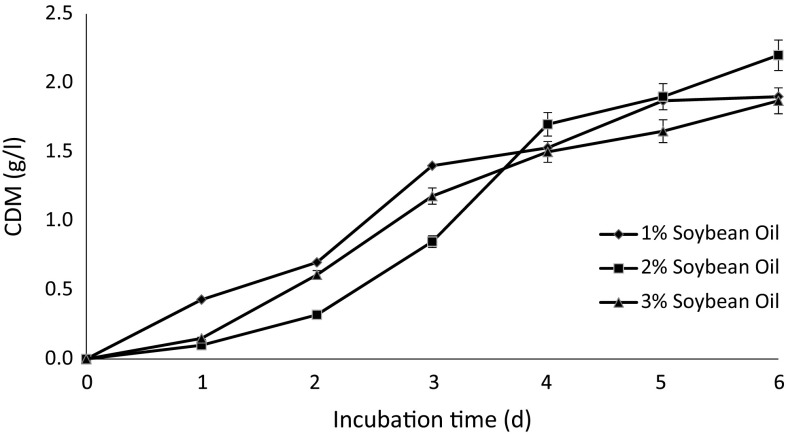
Cell dry mass (CDM) formation during incubation under shake flask conditions

The biomass was measured for the different concentrations of carbon sources fed for bacterial growth. The maximum CDM achieved was 1.9, 2.2 and 1.87 g/l for 1, 2 and 3 % (v/v) of soybean oil, respectively (Fig. 2). With each passing day, the CDM increased irrespective of the concentration of the soybean oil though the relationship was not linear, the maximum value was observed on the 6th day of incubation. The optimum concentration was determined to be 2 % (v/v) on the basis of the results as described in Fig. 2. On moving from 1 to 2 % (v/v) concentration of soybean oil, the CDM increased but it decreased when 3 % (v/v) soybean oil was used. This finding is in accordance with the work previously published on *P. aeruginosa* where increasing the concentration of soybean oil led to the death of microorganism (Khandpur et al. 2012). Different strains of *Pseudomonas* have been reported for PHA production using oily substrates. For instance, 5.8–9.7 g/l of CDM has been observed in *P. aeruginosa* 47T2 (Haba et al. 2007), ~4.3 g/l of CDM up to 72 h using *P. aeruginosa* NCIB 40045 strain (Fernandez et al. 2005), 2.7 g/l of CDM using *P. stutzeri* 1317 strain on soybean oil (He et al. 1998) and by using *P.* *mosselii* TO7 CDM ranging from 1.12 to 4.31 g/l (Chen et al. 2014).

### Production of PHA

**Fig. 3:**
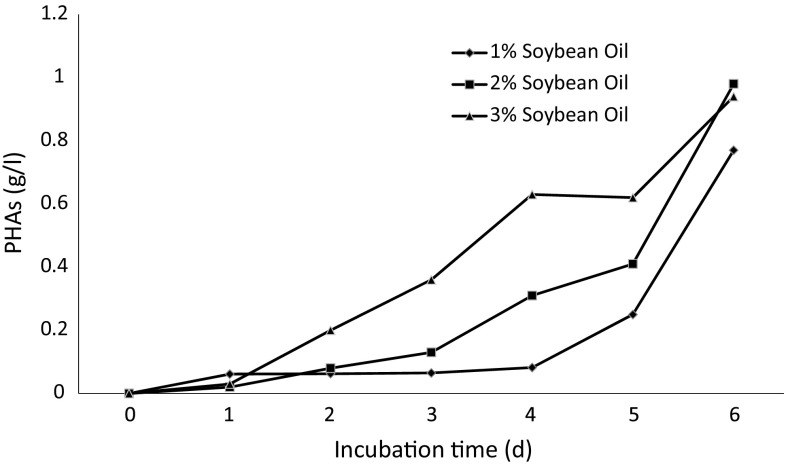
PHAs production during incubation under shake flask conditions

The PHAs from cellular biomass was extracted by solvent extraction and PHA yield was calculated in g/l as shown in Fig. 3. The yield of PHA varied for each concentration of carbon source under study. A samimilar increasing trend was observed with all three concentrations. With each passing day, the PHAs accumulation increased from 1st to the 6th day of incubation. As the time period passed, the bacteria could had consumed nitrogen thus C/N increased which resulted in higher accumulation of PHAs within cells. Maximum PHAs yield of 0.98 g/l was obtained on 2 % (v/v) soybean oil as sole carbon source followed by 3 % (v/v) soybean oil. PHAs accumulation on biomass also varied, the batch with 3 % (v/v) soybean oil gave maximum PHAs accumulation of 50.27 % (w/w) of CDM. This might be due to the fact that PHA accumulation on biomass increased under nitrogen limitations in accordance of already reported work (Basak et al. 2011). The PHAs accumulations of 1, 2 and 3 % (v/v) soybean oil batches were 40.53, 44.55 and 50.27 % (w/w) of CDM, respectively. This could be attributed to the increased value of C/N ratio as the concentration of soybean oil was increased. The increased C/N is known for enhancing the PHAs accumulation in cells (Durner et al. 2001; Lee et al. 2008; Sudesh et al. 2000). Using oils as carbon source, different reports showed different results with respect to PHAs accumulation in cell biomass; PHAs accumulation up to 36 % (w/w) of CDM using *P. aeruginosa* strain (Haba et al. 2007), up to 63 % (w/w) of CDM using *Pseudomonas. stutzeri* strain (He et al. 1998), from 40 to 50 % (w/w) PHAs accumulation of CDM using different triglyceride substrates while 44.5 % (w/w) PHAs accumulation of CDM when grown on soybean oil using *Pseudomonas. resinovorans* (Ashby and Foglia 1998).

### Production kinetics of fermentation

Kinetic parameters of PHAs production using culture of EBN-8 on different concentrations of soybean oil are shown in Table 1. The bacteria grew and produced PHA on all different concentrations of soybean oil used. After 24 h of incubation, the biomass began to increase and reached maximum value on the 6th day of incubation in all samples, though the per day increment was different with all three concentrations. The EBN-8 mutant showed the best growth yield coefficient (*Y*_X/S_) of 0.369 g biomass per gram of soybean oil for both 2 and 3 % (v/v) soybean oil cultures. The value of *Y*_X/S_ increased as the concentration of soybean oil was increased from 1 to 2 and then 3 % (v/v). The product yield related to substrate (*Y*_P/S_) was different for all concentrations and maximum value of *Y*_P/S_ was 0.185 g product per gram of substrate when 3 % (v/v) soybean oil was used for growth, while 2 and 1 % (v/v) soybean oil cultures showed only *Y*_P/S_ of 0.164 and 0.098 g/g, respectively. Maximum product yield with respect to biomass (*Y*_P/X_) was 0.503 g/g achieved on using 3 % (v/v) soybean oil followed by 2 % (v/v) soybean oil as 0.445 g/g, while only 0.405 g/g value was observed using 1 % (v/v) soybean oil and this could be attributed to the C/N ratio increment which plays a significant role in PHA accumulation (Lee et al. 2008; Singh Saharan et al. 2014) and as concentration of soybean oil was increased from 1 to 2 and 3 % (v/v) soybean oil, the C/N ratio also increased favoring more PHA accumulation hence *Y*_P/X_ increased. Maximum volumetric productivity (*P*_V_) of 0.0068 g/l/h was achieved on 2 % (v/v) soybean oil cultures followed by 0.0065 g/l/h on 3 % (v/v) soybean oil cultures which might be due to the less quantity of biomass (1.9 g/l) on 3 % soybean oil cultures as compared to the biomass (2.2 g/l) achieved on 2 % soybean oil cultures. The *P*_v_ of PHAs, using different strains of *Pseudomonas* species, reported in the literature are 0.012–0.11 g/l/h (Tripathi et al. 2011) and 0.004 g/l/h (Kenny et al. 2008). Muhr et al. (2013a) reported *P*_v_ of 0.036–0.067 g/l/h from *P. citronellolis* grown on animal derived waste at bioreactor level. The reason of low *P*_v_ of the present study might be the difference of fermentation mode (batch vs fed-batch) but we observed significantly higher values of PHA accumulation of CDM (50.27 % w/w) as compared to the CDM (9.2 % w/w), achieved by Muhr et al. (2013a).

**Table 1:** Kinetic parameters of PHA production by *P. aeruginosa* EBN-8 on soybean oil

Carbon source level	*Y*_X/S_ (g/g)	*Y*_P/S_ (g/g)	M PHA/CDM (g/g)	*P*_V_ (g/l/h)
1 % soybean oil	0.243 ± 0.024	0.098 ± 0.009	0.405 ± 0.040	0.0053 ± 0.005
2 % soybean oil	0.369 ± 0.037	0.164 ± 0.016	0.445 ± 0.044	0.0068 ± 0.006
3 % soybean oil	0.369 ± 0.037	0.185 ± 0.018	0.503 ± 0.050	0.0065 ± 0.006

### Chemical analysis of the product

**Fig. 4:**
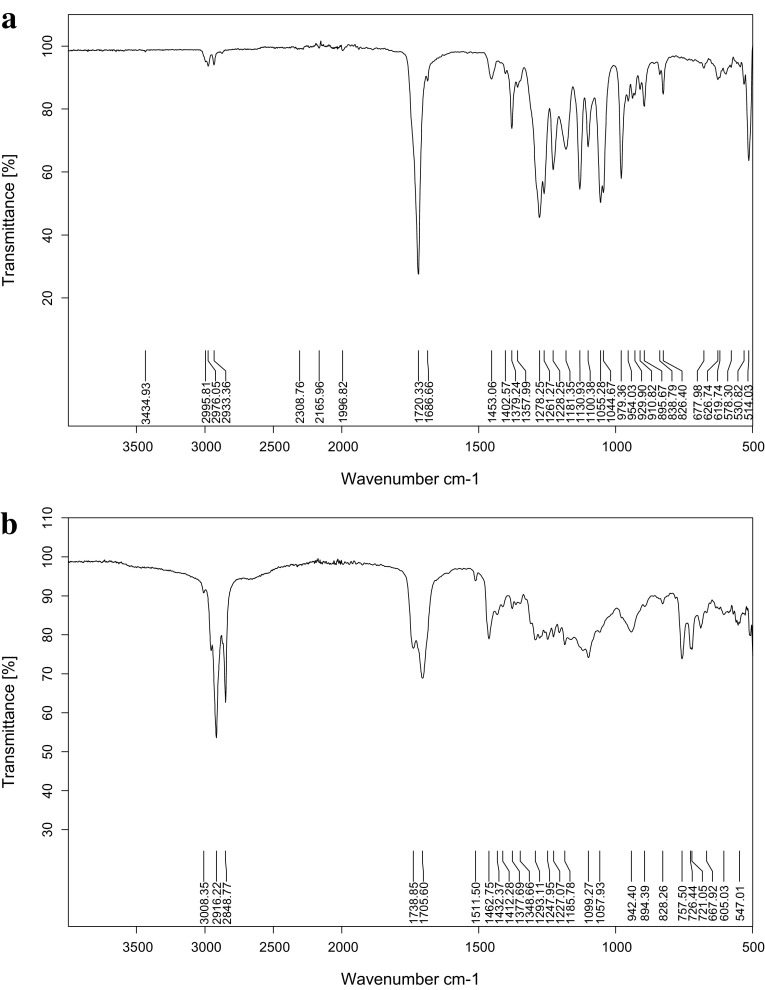
FTIR spectra of **a** PHB and **b** PHA produced by *P. aeruginosa* grown on *n*-hexadecane as carbon source

**Fig. 5:**
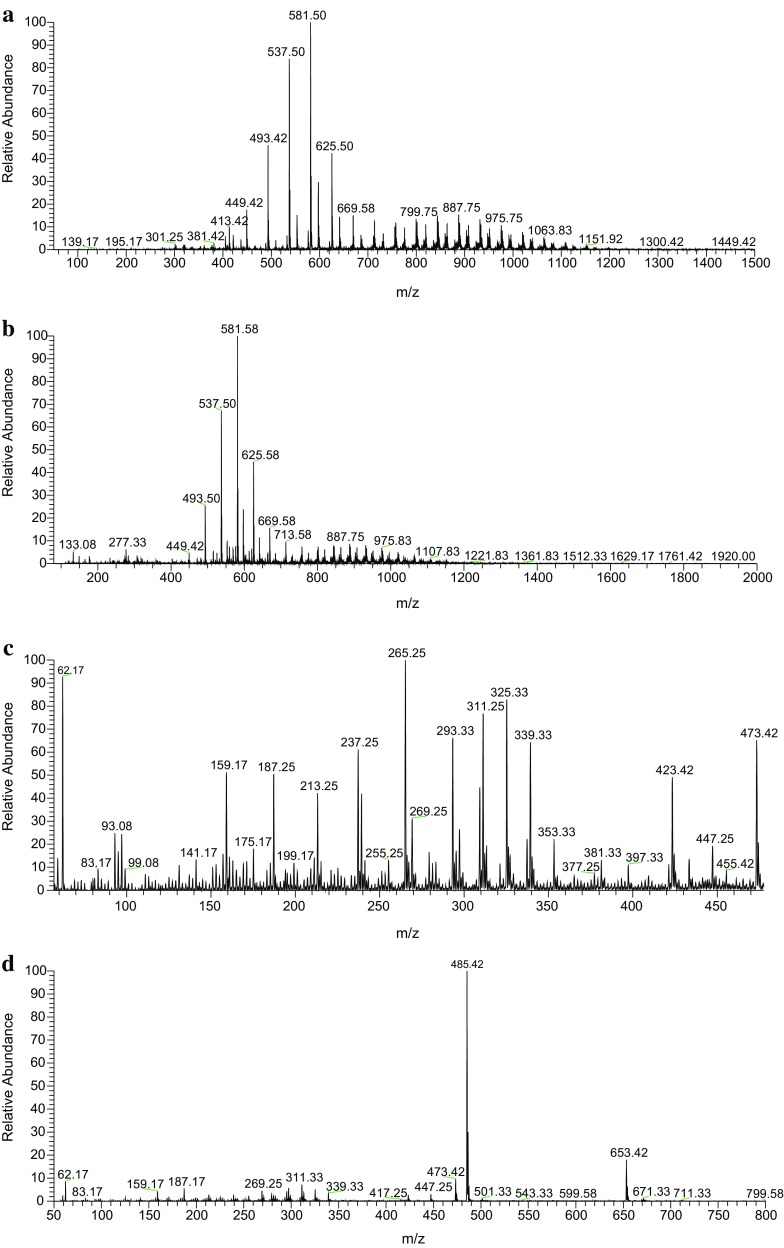
Representative LC–ESI–MS positive mode spectra of **a** PHB and **b** produced PHA; and respective negative mode spectra of **a** PHB and **b** produced PHA

For the confirmation of the extracted polymer, it was checked against standard PHB purchased from Sigma-Aldrich^®^. The FTIR spectra of the standard PHB and extracted PHAs are shown in Figs. 4 and 5, respectively. The standard PHB showed peaks at 1720.33 cm^−1^ which showed the presence of ester group (C=O) in the backbone of the structure and absorption range from 1053 to 1280 cm^−1^ showed the valance vibrations of the carbonyl group as reported in the literature (Biradar et al. 2015). The spectra of extracted PHAs showed peak from 1705.60 to 1738.85 cm^−1^ which represented ester group in the structure.The peaks in the range of 2848.77–3008.35 cm^−1^ showed the presence of CH_3_–, –CH_2_– –CH_2_–, –CH_2_–CH_3_ (Gumel et al. 2014). The absorption at 1377.69 cm^−1^ could be attributed to the terminal CH_3_ groups present as mentioned in the literature at 1378.83 cm^−1^ (Vishnuvardhan Reddy et al. 2008).

The FTIR analysis confirmed the peaks near 1738.85, 2916.22, 2848.77 and 1099.27 cm^−1^ that concluded that produced biopolymer was mcl-PHA copolyesters due to the major difference in peaks near 2900 cm^−1^ of pure PHB and biosynthesized PHAs (Hong et al. 1999; Khare et al. 2014; Kim et al. 2007).

The LC–ESI–MS analysis of the PHA showed a polymer with the molecular mass of *m/z* 448.5 (Fig. 5). Molecular mass of terminal groups was calculated to be 104.5 and that of monomer unit it was 86. The degree of polymerization of produced PHB was observed as 4. Hence, the net molecular mass of polymer was calculated by using the following expression:

3 $${\text{Mol.}}{\text{mass\,of\,PHA}}=104.5+86.0{\times}4 = 104.5 + 344 = {\mathbf{448.5}}\,{\text{amu}}.$$

Hence, the molecular mass of produced PHA was found to be 448.5, due to positive ionization phenomenon of mass spectra proton of both standard PHB and produced PHAs molecules yielded [M + H]^+^ cations at *m/z* 449.5 which can be seen from the spectra giving the confirmation of the compound (Fig. 5a, b). Whereas, the negative mode ESI–MS of both standard PHB and produced PHAs molecules yielded [M−H]^−^ anions at *m/z* 447.5 which can be seen from the spectra giving the confirmation of the compound (Fig. 5c, d). Hence, confirming that the polymer has four basic repeated units linked in a chain forming the PHB structure. *Pseudomonas aeruginosa* has been reported already for the production of PHB (Ayub et al. 2004; Lopez et al. 2009). The mass spectra also showed some other peaks which indicated that other monomers apart from PHB are also present, which require further characterization in future by GC–FID and GC–MS.

## Conclusion

In the present report, polyhydroxyalkanoates production and optimization was done using a *Pseudomonas aeurginosa* strain grown on different concentrations of soybean oil. Maximum yield of polyhydroxyalkanoates as 0.98 g/l and the maximum volumetric productivity as 0.0068 g/l/h were achieved on 2 % (v/v) soybean oil cultures, while the maximum polyhydroxyalkanoates accumulation percentage 50.27 % (w/w) of cell dry mass achieved was on 3 % (v/v) soybean oil culture. The cultures with 3 % (v/v) soybean oil also showed the maximum product yield per gram of substrate and biomass which were 0.185 and 0.503 g/g, respectively.

### Future work

The selected gamma ray mutant of *Pseudomonas aeurginosa* showed good biosynthesis of polyhydroxyalkanoates under shake flask conditions when grown on soybean oil. The strain can be switched up on fermentor (large scale) for the production of polyhydroxyalkanoates, which can be optimized further to achieve economical polyhydroxyalkanoates production. Furthermore, the characterization of the produced biopolymers with GC–FID and GC–MS for monomeric composition analysis.
